# Evaluation and Cross-Comparison of Lexical Entities of Biological Interest (LexEBI)

**DOI:** 10.1371/journal.pone.0075185

**Published:** 2013-10-04

**Authors:** Dietrich Rebholz-Schuhmann, Jee-Hyub Kim, Ying Yan, Abhishek Dixit, Caroline Friteyre, Robert Hoehndorf, Rolf Backofen, Ian Lewin

**Affiliations:** 1 Department of Computational Linguistics, University of Zürich, Zürich, Switzerland; 2 European Molecular Biology Laboratory, European Bioinformatics Institute (EMBL-EBI), Wellcome Trust Genome Campus, Hinxton, Cambridge, United Kingdom; 3 Department of Genetics, University of Cambridge, Downing Street, Cambridge, United Kingdom; 4 Albert-Ludwigs-University Freiburg, Fahnenbergplatz, Freiburg, Germany; University of Illinois-Chicago, United States of America

## Abstract

**Motivation:**

Biomedical entities, their identifiers and names, are essential in the representation of biomedical facts and knowledge. In the same way, the complete set of biomedical and chemical terms, i.e. the biomedical “term space” (the “Lexeome”), forms a key resource to achieve the full integration of the scientific literature with biomedical data resources: any identified named entity can immediately be normalized to the correct database entry. This goal does not only require that we are aware of all existing terms, but would also profit from knowing all their senses and their semantic interpretation (ambiguities, nestedness).

**Result:**

This study compiles a resource for lexical terms of biomedical interest in a standard format (called “LexEBI”), determines the overall number of terms, their reuse in different resources and the nestedness of terms. LexEBI comprises references for protein and gene entries and their term variants and chemical entities amongst other terms. In addition, disease terms have been identified from Medline and PubmedCentral and added to LexEBI. Our analysis demonstrates that the baseforms of terms from the different semantic types show only little polysemous use. Nonetheless, the term variants of protein and gene names (PGNs) frequently contain species mentions, which should have been avoided according to protein annotation guidelines. Furthermore, the protein and gene entities as well as the chemical entities, both do comprise enzymes leading to hierarchical polysemy, and a large portion of PGNs make reference to a chemical entity. Altogether, according to our analysis based on the Medline distribution, 401,869 unique PGNs in the documents contain a reference to 25,022 chemical entities, 3,125 disease terms or 1,576 species mentions.

**Conclusion:**

LexEBI delivers the complete biomedical and chemical Lexeome in a standardized representation (http://www.ebi.ac.uk/Rebholz-srv/LexEBI/). The resource provides the disease terms as open source content, and fully interlinks terms across resources.

## Introduction

Biomedical research has developed into a data-driven scientific domain that profits from knowledge discovery methods [1]. Data interoperability is paramount to make efficient use of the public distribution of resources and to achieve assessment of proprietary data against public content with little overhead costs. In the best case, data integration uses the model of a virtual knowledge broker which benefits from public standards and would also include the scientific literature [2].

### “Lexeome”: the Biomedical Term Space

In particular the integration of the scientific literature with the biomedical data resources requires specific semantic resources that normalize entity mentions, i.e. protein and gene names (PGNs), diseases, chemical entities, to database entries [3]. More in detail, standardized terminological resources such as the BioLexicon contribute to the integration work, since they give an overview on the scope of biomedical terminologies and enable the transformation of the literature into a standardized representation, where the database content is aligned with the literature semantics [4]. In other words, the literature conveys database semantics through referencing of database entries (called “semantic enrichment of the scientific literature”).

The full set of biomedical entities, their baseforms and term variants would form the term space (the “Lexeome”). Certainly the number of terms should correlate with the number of entities and the number of names should be limited, if the number of entities is. On the other side, the proportion of synonyms per entity is unknown, and also the relevance of terms from one domain to another has not been explored, although it can be expected that terms will contain references to other terms where possibly both terms may even belong to different semantic domains. For example, “4-hydroxybenzoate polyprenyltransferase” (UniProt:COQ2 YEAST) is an enzyme that requires the substrate “4-hydroxybenzoate” (ChEBI:17879). Many more such references can be expected across the Lexeome as has been demonstrated by the CALBC project [5].

In addition, the researchers in biomedical ontologies have already acknowledged that the compositional representation of concept labels would improve the consistency data representations and the reuse of resources. The decomposition of terms and the post-compositional representation of concepts has in particular been acknowledged for the representation of phenotypes in humans and mice [6]. Studies based on the gene ontology (GO) have shown that a number of compositional structures are quite frequent and that the identification of the compositional patterns can improve the quality of the curation process and the ontological resource overall [7], [8]. At a smaller scale, similar analyses have been applied to the medical terminology to improve its quality, and in principle we could generate tree-based compositional structures to named entities, but would expect a high degree of complexity for the biomedical scientific domain [9], [10].

Nowadays, the baseforms and term variants are provided from scientific databases and - to a lesser degree - from ontological resources, but little attention has been put to the development, description and analysis of the Lexeome. A terminological resource which is expected to cover the Lexeome will deliver a number of advantages. First, novel terms can be assessed against the Lexeome to avoid ambiguity and redundancy; second, compositional terms can be decomposed and analyzed for their expressiveness in comparison to existing concept labels just like post-compositional concept labels; third, terms from the scientific literature can be referenced to one or many existing terms; and finally, the information from the Lexeome can be used to disambiguate existing terms (see Wordnet usage).

#### Referencing data

Reaching beyond biomedical data integration including the scientific literature, recent visionary developments propose to expose results and findings early on as factual statements in a fixed format (“nanopublications”, “proto-ontologies”, “microparadigms”) and where any data set should have the potential to be referenced and reused electronically from any world-wide access point (digital object identifiers, DOIs for data) [11]-[14]. The representation of the data either follows data formats or requires meta-data for the correct annotation of its origins and experimental settings, but then contributes to the generation and evaluation of hypotheses [15], [16]. These requirements initiated the development of terminological and ontological resources, for example the Unified Medical Language System (UMLS) for the clinical and biomedical domain, the development of ontological resources such as the Gene Ontology (GO) for the representation of conceptual knowledge and eventually the generation of semantic resources that span several domains [4], [17], [18].

### Biomedical Data Resources

The biomedical research community has established primary data resources serving as a standard for biomedical-chemical entities and concepts: UniProtKb (http://www.ebi.ac.uk/uniprot) and EntrezGene (http://www.ncbi.nlm.nih.gov/gquery) for protein and gene entities, Interpro (http://www.ebi.ac.uk/interpro/) for protein families, the NCBI (National Center for Biotechnology Information, http://www.ncbi.nlm.nih.gov) taxonomy for species, and ChEBI (Chemical Entities of Biological Interest, http://www.ebi.ac.uk/chebi/) for chemical entities [19]-[22]. The provided names serve as a baseform (and term variants) for the data entry and are frequently reused for alternative data entries within the database (called “ambiguity”) or in other databases (called “polysemy”) [23]. The ambiguous use of gene and protein names (PGNs) for orthologous entities induces confusion, if the species resolution is required, but improves reuse of scientific data and literature across species [24]-[26]. The same is true for the polysemous use of disease terms with reference to a species, e.g. HIV (human immunodeficiency virus) for the virus and for AIDS (acquired immune deficiency syndrome) caused by the virus [5]. The efficient use of terminological resources helps to mimic human understanding through resolving conflicting interpretations [4].

#### Available terminological resources

It is a common approach that a researcher in the text mining domain would collect terms from the described resource, extract the terminology and would use it for text mining or any other data analysis [27]-[31]. A common format to the different resources and the integration of the terms across the resources would contribute to the interpretation of research results, if the resource has been used in the research [4], [32].

Terminological resources have been proposed for the medical domain, i.e. resources such as the MetaThesaurus and the collection of resources in UMLS [17]. The former is geared towards natural language processing solutions in the medical domain and provides linguistically relevant information. The latter collects and distributes a large number of terms from different resources, but does not integrate them consistently across resources nor resolves the diversity in the license agreements to a unified model. In particular, the use of the biggest and most relevant disease terminology, i.e. Snomed-CT, is limited, since the license agreement allows broad usage only in selected countries which do cover the costs of the country-wide license agreement.

Ontological resources are openly available from the Foundry of Open Biomedical Ontologies, but these resources represent conceptual knowledge in contrast to entity representation as delivered from the biomedical data resources [33]. The BioThesaurus has been produced to gather all PGNs and has developed into a comprehensive resource, however other biomedical and chemical entities are not covered and even the references to enzyme databases and protein families have not been included [32]. The BioLexicon collects terms from different resources and harmonizes the representation, but does not interlink the entities nor includes statistical information from the term usage across literature resources [4]. Jochem is a collection of chemical terms and again the interlinking and cross-comparison with other data resources has not been performed [34].

Additional semantic and terminological resources have already been provided for other domains, for example Wordnet for general English use and Bablenet for multilingual use [35], [36]. Both enable researchers to develop information technology that can deal efficiently with natural language, but neither one is designed to support biomedical applications. Altogether, several resources are in place for different tasks, but a comprehensive standardized terminological resource has not yet been produced in the biomedical domain that gives insights in the distribution and usage of the existing terms.

### Requirements for a Terminological Resource

A terminological resource in the biomedical domain has to integrate semantic types such as genes/proteins, chemical entities, species, diseases and others. Furthermore, it has to cope with complex constructs, since the scientific language anticipates naming for such constructs combining entities of different semantic types and occasionally uncommon syntactic structures. For example, the “Bovine Viral Diarrhea Virus E2 protein” (UniProt:A8VM04 BVDV) is a protein that is induced by a virus (UniProt:POLG BVDVN) that infects the intestinal mucosa of its host organism, i.e. the bovine. As a response, we postulate, that it is paramount to gather all relevant biomedical terms (the “Lexeome”), decompose their syntax and their complex semantic structure, i.e. their nestedness and ambiguities, gather information about their use, and engineer a novel terminological resource that can serve as a hub for modern language processing techniques and data integration solutions connecting literature with biomedical data. LexEBI is the first solution that would serve this purpose in the biomedical domain.

A terminological resource should not only deliver the known terms and the term variants but also additional information, for example information about the usage of terms in the literature or references to related terms, such as different meanings to the same term or the occurrence of a term as part of another term. The BioLexicon is a terminological resource that does deliver a wide range of terms, but is not complete, i.e. lacking medical terms and a significant portion of the chemical terms, and also not cross-referenced between all resources, whereas the BioThesaurus only contributes PGNs [4].

LexEBI contains (1) the full scope of biomedical-chemical relevant terms, (2) abbreviations and their long forms from the scientific literature, and (3) frequency information from the scientific literature. All terms have been cross-compared across the different resources and cross-references are provided as part of the terminological resource.

The terminological resource provides valuable services to the text mining and data integration community.

## Methods

### Publicly Available Resources

LexEBI has been generated from a number of resources that deliver terms or literature content. Two different versions of LexEBI are available that exploit Biothesaurus 6.0 “GP6”; distribution from June 1, 2009) and Biothesaurus 7.0 (“GP7”; June 29, 2010) [32]. We took both versions of the Biothesaurus into consideration, since they differ in their content. Our comparison leads to an improved understanding how complete the compiled resources such as the Biothesaurus are with regards to the contained entities: the smaller resource may be more concise and the larger resource may contribute more term variants of lesser importance. For example, GP7 is larger than GP6 but the increase in size is mainly due to a larger number of term variants which even decreases the performance of PGN tagging solutions [37].

For UniProtKb the release 2010 06 (from June 15, 2010) has been used [19]. Table 1 gives an overview on the overall number of extracted terms. For the literature resources, the British National Corpus (BNC) version 1.0 (released on May 1995) and the PubMed distribution (from Oct 11, 2010) has been used. Interpro version 27, Jochem version 1.0, ChEBI in its release 64 and the release 2010AA of UMLS have been exploited for the presented analyses [34], [38].

**Table 1:** Sources of baseforms and term variants.

		Baseforms [#]	Variants [#]	Total [#]	Total/Labels	Unique terms [#]	Uniq. Terms/Labels
Gene/Prot.	GP7	516′113	4′005′040	**4**′**521**′**153**	8.76	1′726′853	3.35
	GP6	488′577	3′389′316	**3**′**877**′**893**	7.94	1′564′436	3.20
	Interpro	20′671	0	**20**′**671**	1.00	20′671	1.00
	Enzymes	4′905	8′082	**12**′**987**	2.65	12′377	2.52
Chemi- cals	Jochem	278′578	1′691′980	**1**′**970**′**558**	7.07	1′527′752	5.48
	ChEBI	19′645	94′748	**114**′**393**	5.82	101′307	5.16
	ChEBI (all)	549′838	1′187′322	**1**′**737**′**160**	3.16	863′227	1.57
Other	Diseases	56′010	165′581	**221**′**591**	3.96	186′555	3.33
	Species	643′280	199′130	**842**′**410**	1.31	838′135	1.30
UMLS	Pharmact.	104′201	123′840	**228**′**041**	2.19	227′799	2.19
	Bioact.	54′148	87′209	**141**′**357**	2.61	141′121	2.61
	Enzymes	26′065	56′332	**82**′**397**	3.16	82′033	3.15
	Lipid, Carb.	11′518	9′770	**21**′**288**	1.85	21′281	1.85
	Vit., Horm.	6′877	10′258	**17**′**135**	2.49	17′007	2.47
	Neoplast.	4′718	6′488	**11**′**206**	2.38	11′196	2.37

The table shows the distribution of terms from LexEBI sorted according to the resource that delivered the terms. The biggest portions of the terms contained in LexEBI result from BioThesaurus (GP 6 and GP 7), from Jochem and ChEBI and from the NCBI taxonomy. Interpro and species term show a low degree of term variation.

### Extraction of Terms from the Primary Database Resource

The primary resource was processed for the extraction of the contained terms. For the BioThesaurus, the clusters of terms and the term variants were extracted [32]. Terms representing less meaningful names such as “hypothetical gene”, “putative gene”, “probable gene”, “possible gene” and single numbers have been removed, since these terms do not convey any characteristics describing a specific gene or protein entity; they denote sequence similarity between a potentially novel gene and an existing gene. For a detailed description of the morphological features and the semantics of PGNs please refer to [37].

The concept identifiers of each term from each resource have been kept for later reference purposes. The species reference was as well maintained and integrated as a reference to the species mention. All term variants for a given concept have been organised in a single cluster, where the preferred term gives the baseform of the cluster. In the same way, the terms from ChEBI, Jochem, IntEnz, and the NCBI taxonomy have been extracted and processed (see the following example) [39]:

<Cluster clsId = "CHEBI-CHEBI:32" semType = "CHEBI">

<Entry entryId = "CHEBI-CHEBI:32-1" baseForm = "(+)-N-methylconiine" type = "PREFERRED">

<PosDC posName = "POS" pos = "N"/>

<SourceDC sourceName = "CHEBI" sourceId = "CHEBI:32"/>

<Variant WRITTENFORM = "(+)-N-Methylconiine" type = "orthographic"/>

<Variant WRITTENFORM = "(2S)-1-methyl-2-propylpiperidine" type = "orthographic"/>

<Variant WRITTENFORM = "Methylconiine" type = "orthographic"/>

<Variant WRITTENFORM = "C9H19N" type = "orthographic"/>

<Variant WRITTENFORM = "CCC[C@H]1CCCCN1C" type = "orthographic"/>

<Variant WRITTENFORM = "InChI = 1/C9H19N/c1-3-6-9-7-4-5-8-10(9)2/h9H,3-8H2,1-2H3/t9−/m0/s1"

type = "orthographic"/>

<DC att = "KEGG COMPOUND accession (KEGG COMPOUND" val = "C10159)"/>

<DC att = "CAS Registry Number (KEGG COMPOUND" val = "35305-13-6)"/>

<DC att = "CAS Registry Number (ChemIDplus" val = "35305-13-6)"/>

<DC att = "Beilstein Registry Number (Beilstein" val = "79936)"/>

<Relation type = "has-parent-hydride" target = "CHEBI-CHEBI:28322"/>

</Entry>

</Cluster>

Furthermore, the UMLS terminological resource has been processed to extract relevant terms characterizing protein, gene and chemical entities. The terms have been filtered using their type assignments and terms from the following categories have been extracted: (1) antibiotic and neuroreactive substances, (2) biologically active substances, (3) enzymes, (4) lipids and carbohydrates, (5) pharmacological active substances, and (6) vitamins and hormones. Other categories such as disease and disorder and immunological factors have been ignored. The order of categories has been applied, if one category had to be selected from a dual assignment. Our manual evaluation ensured coherence across the selected categories. The cross-comparison of chemical entities and proteins/genes against these categories gives a categorization of terms according to UMLS and can be exploited whenever named entities have to be interpreted for a particular biomedical reason, e.g. as a lipid or a hormone.

### Extraction of Disease Terms from Medline

Medline is a rich source of disease terminology that can be made publicly available in contrast to standard resources that are only available upon proper licensing. Alternative resources are either not freely available, such as Snomed-CT, or are very limited in their content, such as the disease ontology [40], [41]. To extract the disease terminology from the Medline distribution, the text has been processed to identify stretches of text that contain words that have been identified in a disease terminology. All chunks have been stemmed, normalized and indexed using Lucene [42]. For a given term, the chunk has been processed with MetaMap to assign the concept identifier and compared against the UMLS resource [43].

Terms from Medline that do not appear in the primary terminological resource have been normalized. Different orthographic variants have been identified and then normalized to a single base form, i.e. to the same concept unique identifier (CUI) for a single cluster that has been automatically derived from an UMLS term; but not necessarily representing any exact string in UMLS. Orthographic variation includes variation in upper and lower case in particular in capitalizations, minor changes to the punctuation such as the use or lack of hyphens, the identification and normalization of plural forms, and the different variants of Greek letters.

WordNet has been used to accept chunks that could represent synonyms of terms, i.e. “liver sarcoma” was accepted as a synonym for liver cancer and assigned an UMLS identifier since “sarcoma” is a synonym to “cancer” according to WordNet and “liver cancer” has been retrieved from UMLS. Note that “sarcoma” is a more specific type of a cancer and therefore “sarcoma” is a narrower synonym for “cancer”. As a result, LexEBI delivers term variants to disease terms where the term variant stems from Medline and is fully referenced to UMLS.

### Identification of Acronyms and Long-forms from the Scientific Literature

Acronyms are a very interesting set of terms from the scientific literature: they represent terms with high relevance for a given document and - for a smaller number of established acronyms - expose standardized semantics across the whole scientific literature, e.g. DNA (deoxyribonucleic acid) and HIV. They form a set of terms that is not a priori linked to a predefined semantic type - other than chemical entities which can be identified by their syntactical structure or PGNs which have an overrepresentation of specific terms - but they still enable the attribution of a semantic type through the long form of the acronym definition. We use this resource as a means to determine the representation of the different semantic types across the scientific literature.

For our analyses we have extracted acronyms that have been referenced together with their long form in the scientific literature, i.e. in Medline abstracts and in PubmedCentral full text documents [44]. We identified the following two syntactical structures „abbreviation (long form)“ or” “long form (abbreviation)” using Schwartz-Hearst language patterns which have been evaluated and shown to reach an F1-measure of about 89% [45]. Nonetheless, further research has shown that higher performances can be reached by applying machine-learning solutions either for the acronyms alone (BioADI, up to 90%) or the pairs composed of the abbreviation and its long-form (up to 91% for Ab3P; 91.4% from Yeganova et. al), which was not relevant for our rather limited experiments [46]-[48].

In total we collected 2,016,822 unique abbreviations from 9,969,109 occurrences in overall 11,187,291 Medline abstracts. Note, that a single unique abbreviation can be categorized to two or more different semantic types. For example, LPS is an abbreviation representing the baseform “lipopolysaccharide”, and is linked to entries in ChEBI as well as to entries in GP6 or GP7. The distribution of the abbreviations across the different data resources is shown in table 2. All abbreviations have been matched to the term entries from the different term repositories, i.e. to ChEBI entries or to UniProtKb entries, according to their long-form (LF) that has been mentioned in combination with the short-form in the literature. The frequency of the identified abbreviation-LF pairs has been identified across the whole literature resources, i.e. across BNC and Medline. The abbreviations have been matched against the term variants from the other data resources to identify their terminological counter-parts and interlink them with the other data types. An acronym cluster in LexEBI refers to the long form representation and carries the ACRO tag for identification.

**Table 2:** Distribution of abbreviations.

	All occ. in Medline [#]	Unique acronyms [#]	Occ./acronym	>1 occ. [#]	>1 occ. [%]
GP7	1′705′358	65′674	26.0	33′031	49.7%
Enzymes	287′219	10′001	28.7	5′184	48.2%
ChEBI	7′411′169	27′776	266.8	14′080	49.3%
Disease	9′034′479	25′377	356.0	13′610	46.4%
Species	218′964	10′373	21.1	4′670	55.0%
**Total**		**139**′**201**		**70**′**575**	

The abbreviations extracted from Medline have been attributed to a reference terminological resource, e.g. ChEBI, and the frequency of the abbreviation has been determined and added to LexEBI. Half of the abbreviations have single occurrences.

### Cross-comparison and Statistics

The terminological resources have undergone cross-comparison using different comparison methods. First, the terms have been compared using exact matching. As an alternative, morphological variation was included (called “fuzzy match”) that ignored the following variants: First, in the case of a mixed-case term representation with the initial letter in upper-case, the initial letter is also matched against the lower-case variant and vice versa (e.g. Raf vs. raf). Second, a gene or locus named after its associated phenotype may contain the characters of a dash or slash to indicate the wild type or mutant allele, and hence both characters are biologically meaningful, but in other cases they are used synonymously for white space and thus have been ignored during the matching.

**Figure 1:**
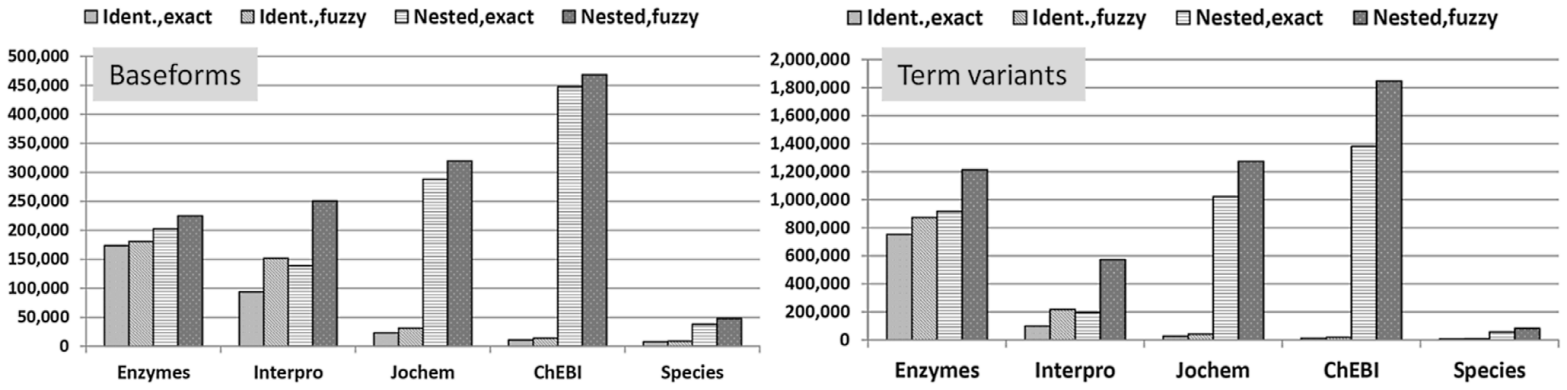
Baseform polysemy and nestedness: The diagram shows several comparisons between the different data resources. The content of the mentioned five resources, i.e. Enzymes, Interpro, Jochem, ChEBI and Species, against the terms contained in GP7 using exact matching and fuzzy matching that considers morphological variation. All comparisons only use the baseforms of the clusters in LexEBI (left part) or the term variants from different resources (right part). The measurements have been performed for the identification of complete terms in the resource and for the nestedness of GP7 terms in the terms of the other resources, i.e. “Identical” versus “Nestedness”, respectively. It can be seen, that terms denoting enzyme entities do not show extensive term variation in GP7 and are nested to only a small extent in other terms of GP7. On the other hand, the terms for chemical entities are nested to a large extent in the terms of GP7 forming the cause of ambiguity and nestedness. Again the terms from Jochem and from ChEBI are part of the term variants from GP7 using exact matching and matching based on morphological variation.

Last, for nested tagging, one terminological resource contributed the nested terms and the other one was used for being tagged, i.e. the nested terms have been tagged inside of the tagged terms - again applying either exact or fuzzy matching. The nestedness of a term gives an indication to which extent one terminological resource has a compositional structure that relies on another resources, and possibly even another semantic type. Fig. 1 displays the tagging of terms from different resources, e.g. enzymes, Interpro and chemical entities against GP7 using either the PGN baseforms (left diagram) or the term variants (right diagram).

### Calculating Statistical Parameters Across Medline and BNC

The terminological resources have been used to annotate entities across the whole content of Medline to identify the term frequencies; the same approach has been applied to BNC as well. The availability of term frequencies in the different corpora offer the opportunity to disambiguate terms based on their frequencies in a medical and a general English corpus and - at a later stage - the frequency parameters can be integrated as a discriminatory feature into basic disambiguation techniques or into machine-based classification methods [37]. For example, “water” is a chemical entity (CHEBI:15377) and an unspecific term, i.e. it would show high frequencies in Medline and in BNC, whereas Oxytocin (CHEBI:7872, UniProtKb:NEU1 HUMAN, HGNC:8529) is a very specific term and would only show in the medical literature a high term frequency.

### Term Representations in the Terminological Resource

LexEBI uses an XML format for the representation and storage of the terminological resource (see method section). Explicit reference are implemented to the preferred term, the term variants, concept identifiers, term frequency in the BNC, in Medline, and the frequency of the term variants. An additional table makes reference to the nestedness of the terms in the resources.

## Results

LexEBI collects terms from different public resources and combines them with the help of a standardized format. Furthermore, cross-references have been established between related data entries to support identification of polysemous terms and to make use of different interpretations of a given term. Statistical information about the use of terms in different public literature resources has been added to the data entries. This information can be used to distinguish specialized terms from common English terms [37]. Last, the references to biomedical data resources are kept to enable exploration of additional information linked to the data entries.

In the following sections we will explore the distribution of terms to derive key parameters from the terminological resources describing the Lexeome.

### Distribution of PGNs in LexEBI

The terminological resource LexEBI contains 2,729,134 clusters that make reference to a baseform, 13,598,649 term variants and 5,791,531 unique terms in total, where double mentions of the same term (“redundancy”) have not been removed between the different resources (cf. table 1).

For the terminology linked to genes and proteins, two different resources of the same origin have been analyzed, i.e. Biothesaurus 6.0 (called “GP6” for Gene/Protein-6) and the next version, i.e. Biothesaurus 7.0 (called “GP7”). The reason for this comparison is the assumption that the evolution of such semantic resources show growth only to a very limited extent, since the number of entities represented by a term and relevant to the biomedical domain is limited, and it takes time to explore and find novel entities through basic research.

In addition, it is important to characterize the differences between terminological resources, e.g. between GP6 and GP7 and between ChEBI and Jochem, since we do know that a larger terminological resource, e.g. for PGNs, will not necessarily improve the F1-measure of PGN-tagging solutions [37], which is explained by the fact that a conserved portion of PGNs is already included in smaller PGN terminological resources and this part forms - in contrast to a larger number of term variants - the core of the terminological space for PGNs.

GP6 gives access to 1,564,436 terms and GP7 to 1,726,853 terms. 1,444,247 are shared between both resources using exact matching. This results to 92.3% of the unique terms in GP6 and to 83.6% in GP7, showing that the new version contains a larger number of terms and some terms from the older version have been removed (overall growth rate less than 10%).

#### PGN variation

When using terminological variation in the comparison, we determine that 1,549,890 (99.1%) of the terms in GP6 can already be matched with the content of GP7, whereas only 1,641,926 (95.1%) of the terms in GP7 can be matched using the content of GP6. This shows that additional term variants have been added to GP7 that show greater morphological variation than the usual morphological variation of genes and proteins. In other words, GP6 covers already a complete version of the terminology related to gene and protein mentions: in total, GP7 contains 27,536 additional clusters or baseforms that account for 162,417 additional unique terms and 643,260 overall term variants (including redundancy).

The terminological resources for genes and proteins show a high number of term variants per cluster, i.e. 8.76 and 7.94 for GP7 and GP6, respectively, and also high numbers of term variants for chemical entities, i.e. 7.07 and 5.82 for Jochem and for ChEBI. Term variation is only of minor importance for species terms (1.31) and for the other resources.

### Cross-comparison of Semantic Types Across LexEBI

The content from LexEBI has been analyzed in several forms: (1) the terminological resources have been evaluated against each other to quantify polysemous and nested use of terms across terminological resources, and (2) the terms have been extracted from the public scientific biomedical literature to determine the use and distribution of terms in written text.

The degree of polysemous use of terms helps to disambiguate terms at a later stage and in the case of nestedness of terms; we can identify the compositional structure of terms and exploit it for the identification of terms. It can be expected that nestedness occurs more frequently between chemical entities and PGNs and between species and PGNs, but at a lower rate between diseases and chemical entities. The resolution of such nested terms offers new ways of interpreting the terms. More in detail, we would expect that we do not only assign a single label to a term, but would be able to assign labels to its components and eventually read terms similarly to event representations. After all, such an interpretation of terms could mimic the ways how humans read composite terms and would lead to novel indexing approaches that handle complex semantics (see also MedEvi [49]).

#### Analysing PGNs

Several resources have been compared against GP6 and GP7. For a complete overview please refer to table 3. The table gives an overview on the terms that are shared between different resources. For example, 150,104 enzyme baseforms from the IntEnz database are already covered in GP6 and this number increases to 173,994 for the GP7. Morphological variation only adds little to the identification of terms (157,099 and 180,829), whereas nestedness adds a bigger portion to the number of matched terms leading to now 178,155 and 202,484 terms for exact matching and 200,921 and 224,877 terms for fuzzy matching of contained terms. By contrast, terms from Interpro occur in the GP6 and GP7 at lower numbers, 88,613 and 93,979 for both resources respectively, but the number increases to a considerable degree, if fuzzy matching or nestedness is considered (cf. fig. 1). This shows that the generic terms from Interpro form parts of the terms in GP6 and GP7 in contrast to the terms denoting enzymes.

**Table 3:** Baseforms and term variants of different types contained in GP6 and GP7.

		Against baseforms only								Against all terms							
	Nested	Matching				Nestedness				Matching				Nestedness			
	Term	Exact M.		Fuzzy M.		Exact M.		Fuzzy M.		Exact M.		Fuzzy M.		Exact M.		Fuzzy M.	
**GP6**	Enzymes	150′104	**30.7%**	157′099	**32.2%**	178′155	**36.5%**	200′921	**41.1%**	611′645	**15.8%**	711′603	**18.4%**	739′827	**19.1%**	975′362	**25.2%**
	Interpro	88′613	**18.1%**	134′129	**27.5%**	131′094	**26.8%**	224′739	**46.0%**	92′431	**2.4%**	193′964	**5.0%**	177′819	**4.6%**	477′168	**12.3%**
	Jochem	21′461	**4.4%**	28′911	**5.9%**	253′875	**52.0%**	284′856	**58.3%**	25′033	**0.6%**	39′137	**1.0%**	1′148′792	**29.6%**	1′000′229	**25.8%**
	ChEBI	10′388	**2.1%**	13′090	**2.7%**	411′622	**84.2%**	431′645	**88.3%**	10′950	**0.3%**	16′708	**0.4%**	1′148′792	**29.6%**	1′530′209	**39.5%**
	Species	7′823	**1.6%**	8′820	**1.8%**	36′043	**7.4%**	44′454	**9.1%**	7′871	**0.2%**	9′087	**0.2%**	51′581	**1.3%**	72′848	**1.9%**
**GP7**	Enzymes	173′994	**33.7%**	180′829	**35.0%**	202′484	**39.2%**	224′877	**43.6%**	754′229	**16.7%**	872′211	**19.3%**	918′263	**20.3%**	1′214′663	**26.9%**
	Interpro	93′979	**18.2%**	151′797	**29.4%**	138′979	**26.9%**	250′599	**48.6%**	100′087	**2.2%**	217′898	**4.8%**	197′252	**4.4%**	573′201	**12.7%**
	Jochem	23′402	**4.5%**	31′418	**6.1%**	288′062	**55.8%**	319′561	**61.9%**	27′478	**0.6%**	43′214	**1.0%**	1′023′729	**22.6%**	1′273′755	**28.2%**
	ChEBI	11′053	**2.1%**	14′124	**2.7%**	447′812	**86.8%**	468′723	**90.8%**	11′724	**0.3%**	18′128	**0.4%**	1′381′545	**30.6%**	1′847′750	**40.9%**
	Species	7′884	**1.5%**	9′071	**1.8%**	38′356	**7.4%**	48′419	**9.4%**	7′938	**0.2%**	9′294	**0.2%**	57′469	**1.3%**	83′237	**1.8%**

The reference data resource (“tagged term”) is either GP6 or GP7 and the alternative data resources (“nested term”) are ChEBI, Enzyme, Interpro and other resources.

The percentage indicates, which portion of the terms has been tagged.

The increase in matched terms is even stronger, when matching chemical entity terms from Jochem or ChEBI against the PGNs in GP6 and GP7. This shows that the terms from chemical entities have to be considered as compositional components to the gene and protein names. The same observations are even more prominent, if the term variants have been included into the analysis (cf. fig. 1). Now the number of term variants identified in GP7 increases beyond 600,000 terms for the exact matching, but stays below the full number of term variants linked to GP7; the absolute numbers for the matched term increases but the relative numbers are below the figures achieved against the baseforms only.

This shows that the added variants have a higher diversity than the labels only.

#### Analysing chemical entities

Finally, the inverse comparison has been performed, where terms from LexEBI have been tested for their inclusion as nested terms into the terms denoting for example chemical entities and other types (see table 4). It becomes clear that ChEBI forms a central role in the composition of terms since chemical entities form part of the baseforms of the Interpro terms and the baseforms from the UMLS terminologies.

**Table 4:** Baseforms and term variants of different types contained chemical term resources.

		Against baseforms only								Against all terms							
TaggedTerms	NestedTerms	Matching				Nestedness				Matching				Nestedness			
		Exact M.		Fuzzy M.		Exact M.		Fuzzy M.		Exact M.		Fuzzy M.		Exact M.		Fuzzy M.	
ChEBI	Jochem	13′694	**69.7%**	14′683	**74.7%**	15′929	**81.1%**	17′199	**87.5%**	56′155	**3.2%**	60′332	**3.5%**	76′885	**4.4%**	86′470	**5.0%**
	GP6	1′019	**5.2%**	1′258	**6.4%**	11′148	**56.7%**	12′442	**63.3%**	1′264	**0.1%**	1′711	**0.1%**	25′017	**1.4%**	31′356	**1.8%**
	GP7	1′119	**5.7%**	1′366	**7.0%**	11′690	**59.5%**	12′843	**65.4%**	1′416	**0.1%**	1′879	**0.1%**	27′461	**1.6%**	33′565	**1.9%**
	Enzymes	17	**0.1%**	22	**0.1%**	94	**0.5%**	130	**0.7%**	17	**0.0%**	25	**0.0%**	98	**0.0%**	165	**0.0%**
	Bioact.	479	**2.4%**	669	**3.4%**	1′895	**9.6%**	2′569	**13.1%**	667	**0.0%**	1′218	**0.1%**	3′213	**0.2%**	4′960	**0.3%**
	Enzymes	27	**0.1%**	35	**0.2%**	163	**0.8%**	435	**2.2%**	31	**0.0%**	45	**0.0%**	260	**0.0%**	1′147	**0.1%**
	Pharmact.	2′043	**10.4%**	2′569	**13.1%**	5′648	**28.8%**	7′580	**38.6%**	4′096	**0.2%**	6′096	**0.4%**	12′256	**0.7%**	18′454	**1.1%**
Jochem	ChEBI	15′511	**5.6%**	17′104	**6.1%**	215′072	**77.2%**	256′468	**92.1%**	67′030	**3.4%**	86′499	**4.4%**	668′716	**33.9%**	1′142′849	**58.0%**
	Enzymes	114	**0.0%**	125	**0.0%**	373	**0.1%**	474	**0.2%**	143	**0.0%**	169	**0.0%**	607	**0.0%**	930	**0.0%**
Interpro	GP6	2′542	**12.3%**	3′488	**16.9%**	15′946	**77.1%**	19′888	**96.2%**	2′542	**12.3%**	3′488	**16.9%**	15′946	**77.1%**	19′888	**96.2%**
	GP7	2′565	**12.4%**	3′709	**17.9%**	15′508	**75.0%**	20′108	**97.3%**	2′565	**12.4%**	3′709	**17.9%**	15′508	**75.0%**	20′108	**97.3%**
Enzyme	GP6	2′514	**51.3%**	2′604	**53.1%**	4′334	**88.4%**	4′517	**92.1%**	8′400	**10.2%**	8′690	**10.5%**	11′547	**14.0%**	12′280	**14.9%**
	GP7	2′561	**52.2%**	2′657	**54.2%**	4′391	**89.5%**	4′576	**93.3%**	8′601	**10.4%**	8′872	**10.8%**	11′674	**14.2%**	12′383	**15.0%**
	ChEBI	13	**0.3%**	13	**0.3%**	1′732	**35.3%**	3'270	**66.7%**	13	**0.0%**	13	**0.0%**	3′166	**3.8%**	7′386	**9.0%**
	Jochem	91	**1.9%**	102	**2.1%**	3′042	**62.0%**	3′391	**69.1%**	106	**0.1%**	122	**0.1%**	6′392	**7.8%**	7′744	**9.4%**
Bioact.	ChEBI	432	**0.8%**	579	**1.1%**	41′221	**76.1%**	43′864	**81.0%**	553	**0.4%**	1′295	**0.9%**	85′412	**60.4%**	98′536	**69.7%**
Enzymes	ChEBI	21	**0.1%**	28	**0.1%**	16′834	**64.6%**	18′985	**72.8%**	21	**0.0%**	42	**0.1%**	35′223	**42.7%**	49′917	**60.6%**
Pharmact.	ChEBI	2′342	**2.2%**	2′867	**2.8%**	37′373	**35.9%**	47′049	**45.2%**	3′361	**1.5%**	6′559	**2.9%**	58′178	**25.5%**	86′264	**37.8%**

The table gives an overview on the number of terms from the reference data resource (“tagged term”), e.g. ChEBI, Jochem, that contain the term from the alternative data resource (“nested term”). The percentage indicates the portion of the reference data resource.

The overlap between the resources, i.e. the matching of baseforms and the induced semantic polysemy, remains low. Only enzyme terms are covered from GP7 and GP6 as well as from ChEBI and Jochem. The overlap between ChEBI and Jochem is high by the nature of both resources and remains high when the term variants of both resources are compared (right side of the table).

In total, the content ChEBI is disjoint from the other resources, but also ChEBI terms from part of terms from the other terminological which leads into a good compositional structure of the terminological resources. Enzyme terms form also a unique resource and show little morphological variation. The reuse of enzyme entities in the other terminological resources could be reduced, but does not induce major problems. For Interpro we can identify that it does show significant overlap with GP6 and GP7, which is not unexpected, but it would be advantageous if general Interpro terms, i.e. the protein family terms, would be clearly separate from specific PGNs to reduce hierarchical polysemy.

#### Nestedness of unique terms according to their type

In the previous studies, we ignored the fact that terms, e.g. for protein and gene entities, have been reused for different entities, i.e. ambiguous terms specifying two different entities are redundant in a terminological resource, but redundancy has to be kept to reference all entities through all their synonyms. In this next step, we have reduced redundancy and have again analyzed which terms of a given type are included in terms of other types, e.g. terms for chemical entities form frequently part of a PGN. Initially we compared only the baseforms of the terms from different resources (cf. table 5). From an ideal perspective, we would expect that baseforms are not shared between semantic types to avoid ambiguity in the concept labels. But, this assumption has to be validated and a different result cannot be excluded, since the resources have been developed independently from each other and ambiguity can only be avoided due to interactions between the different development teams.

**Table 5:** Nestedness of terms.

	Nested Term		Chemical		Species		Diso		PGN	
Tagged	Total	Unique	Total	Unique	Total	Unique	Total	Unique	Total	Unique
Term			17′311	926	1′895	307	727	131	169	98
PGN	17′728	15′538	16′328	774	1′109	119	291	55		
Diso	1′894	1′824	983	285	786	195			125	70
Species	435	435					435	82	0	
Chemical	45	45					1	1	44	31

The terms from LexEBI have been cross-compared for the identification of nested terms. The figures in the table have been reduced to the number of those terms that do contain a nested term of a different type. The rows represent the terms that have been hosting other terms (“tagged term”) and the columns indicate the tagging terms that nest inside of the hosting terms (“nested term”). Non-redundant counts (“Unique”) are presented in addition to several mentions of the same term, if it contains different nested terms (“Total”). Please note that table 3 counts a cluster as a single entry even if two clusters share the same baseform whereas this table takes a single term as a single count.

We identified that the baseforms do not suffer from polysemy, i.e. the different terminological resources are disjoint with a few exceptions. This is not anymore true, when taking all the term variants into consideration, and - in addition - we find terms of different types contained in other terms. Table 5 gives an overview of the results.

In total only 774 unique chemical entity terms are nested in 16,328 protein/gene terms whereas only 285 chemical entity terms are contained in only 983 disease terms. Species terms are contained as well in PGNs, although the annotation guidelines suggest that species should not be part of the protein name. Disease terms can be part of PGNs as well as species names indicating that a few terms are ambiguous, i.e. belong to the semantic types of species and disease alike.

Table 6 lists the most frequent nested terms and their frequencies. In general, the semantics of the nested terms is correctly attributed. The chemical entity terms and the PGNs are specific with a few exceptions, i.e. “retinal” and “group” for a chemical entity. The disease terms include a few false positive results (“anterior”, “ganglion”, “sympathomimetic”) and polysemous acronyms (“hip”). The list of species terms shows a high variety including hypothetical false positive results (“Beta”, “cis”, “glycine”, “helix”) which could all be verified as true positive results for a species. Altogether, any solution that would consider the ambiguous or nested use of the presented terms should be able to improve its annotation results, and would produce a term representation that complies with the interpretation of a term by an expert.

**Table 6:** List of nested terms.

	ChEBI
**PGN**	1434/ATP, 1026/threonine, 677/nucleotide, 673/serine, 515/peptide, 472/zinc, 445/inhibitor, 392/phosphate, 380/toxin, 292/glycoprotein, 280/NADH, 265/metal, 250/GTP, 184/UDP, 182/S-adenosyl-L-methionine, 179/amine, 168/leucine, 167/acid, 166/hormone, 157/ADP, 149/quinone, 148/tyrosine, 146/cytochrome P450, 143/amino acid, 138/NAD(P)H
**Diso**	157/drug, 83/retinal, 42/alcohol, 23/steroid, 20/hormone, 14/acid, 13/hemoglobin, 13/glucose, 12/iron, 12/growth hormone, 11/pyruvate, 11/lipid, 11/inhibitor, 11/glycogen, 11/cocaine, 10/potassium, 10/group, 10/cholesterol
	**Species**
**PGN**	469/Beta, 87/Glycine, 86/cis, 45/helix, 39/cancer, 28/glycine, 25/Spea, 24/ammonia, 23/Scolopendra, 20/Squamosa, 18/root, 13/Cancer, 12/iso, 11/anemia, 10/Helix, 8/Paes, 8/mago, 7/transposon Tn4556, 6/prion, 6/Ammonia, 5/Transposon Tn7, 5/codon, 5/Cis
**Diso**	224/cancer, 86/anemia, 44/ataxia, 41/glaucoma, 36/bovine, 29/purpura, 23/root, 14/vertigo, 9/trichophyton, 8/salmonella, 8/agnosia, 7/scleroderma, 7/rosacea, 7/Escherichia coli, 6/trichophyton rubrum, 6/microsporum, 5/fossa, 3/trichophyton verrucosum, 3/trichophyton soudanense, 3/patella, 3/nephroma
	**Diso**
**PGN**	81/Sperm, 44/sperm, 18/Mpe, 17/Neuroblastoma, 14/dissociation, 9/anterior, 7/Wiskott-Aldrich syndrome, 7/Anterior, 6/azoospermia, 5/Epstein, 5/Cat eye syndrome, 4/Ten, 4/sma, 4/ns4, 4/Ifi, 4/homocystinuria, 4/ganglion, 4/defect, 3/Nod, 2/Water stress, 2/Tubulointerstitial nephritis, 2/Teratocarcinoma
**Species**	99/Myrmecia, 55/parvovirus, 31/Sheeppox, 23/dgi1, 22/Vaccinia, 21/E11, 15/Yellow fever, 15/Vesicular stomatitis, 13/Erysipelothrix, 13/Avian sarcoma, 10/melas, 9/Hydrometra, 7/Camelpox, 5/Epstein, 5/Caprine arthritis encephalitis, 5/Canine distemper, 5/Budgerigar fledgling disease
**ChEBI**	1/sympathomimetic
	**PGN**
**Diso**	18/insulin, 16/hip, 8/itch, 4/prolactin, 3/angiotensin converting enzyme inhibitor, 3/agglutinin, 2/trypsin, 2/robin, 2/methylmalonyl coA mutase, 2/gastrin, 2/fibrinogen, 2/beta galactosidase, 2/arylsulfatase, 2/androgen receptor, 2/actin, 1/ubiquitin, 1/tyrosinase
**ChEBI**	4/PAP, 3/thioredoxin, 3/ferredoxin, 2/Trp, 2/L-4, 2/IMP, 2/cholinesterase, 2/adrenodoxin, 2/A14, 1/urease, 1/serine proteinase inhibitor, 1/PNP, 1/phospholipase A2 inhibitor, 1/P2Y2, 1/oxytocin, 1/neuraminidase, 1/NAD(P)H, 1/myoglobin, 1/lipoxygenase, 1/lipopeptide

The table shows the most frequent terms from one type (column labels) that are included in the terms of another type (row labels). Note that disease terms appear as part of a species term, since a disease term with the extension “virus” forms the species term.

### Visualisation of Term Nestedness Per Semantic Type

According to the presented analyses, only a small portion of terms of one type is nested in a larger number of terms of another type. Chemical entities form core elements, PGNs show a high variety and a number of terms are poysemous (or ambiguous) between the species and diseases. To visualize better these results, we have generated graphs for the different semantic types, where the semantic type is color encoded and the inclusion of a term is represented by the “nested-in” relation giving the “graphs of nestedness”.

**Figure 2:**
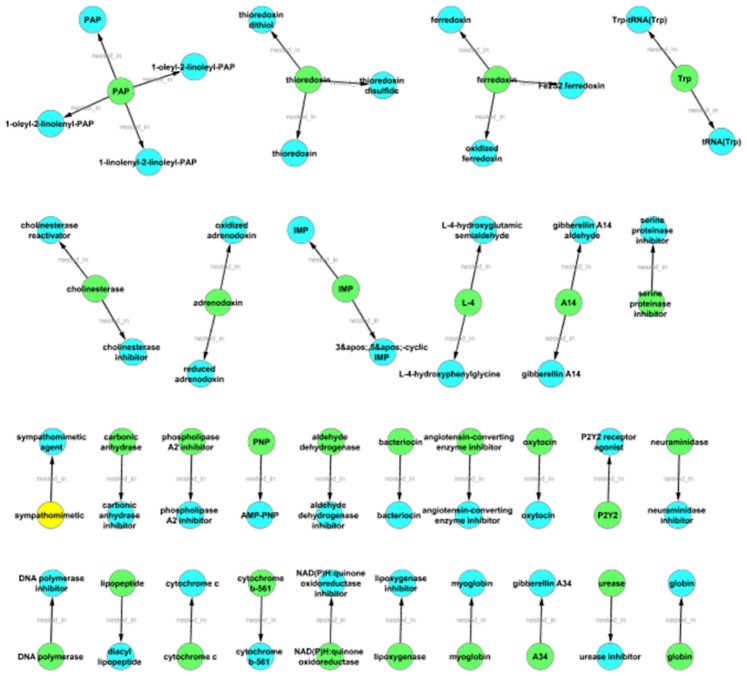
Graphs of nestedness for chemical entity terms: The figure gives an overview on the graphs based on those terms for chemical entities that are composed of a term of a different type. An edge exists between two nodes, if the term from one node is nested in the term of the other node. The color encoding is green for PGNs, red for species, yellow for diseases and blue for chemical entities. Only few terms from ChEBI make use of generalised PGNs in contrast to the nestedness of terms for PGNs.

**Figure 3:**
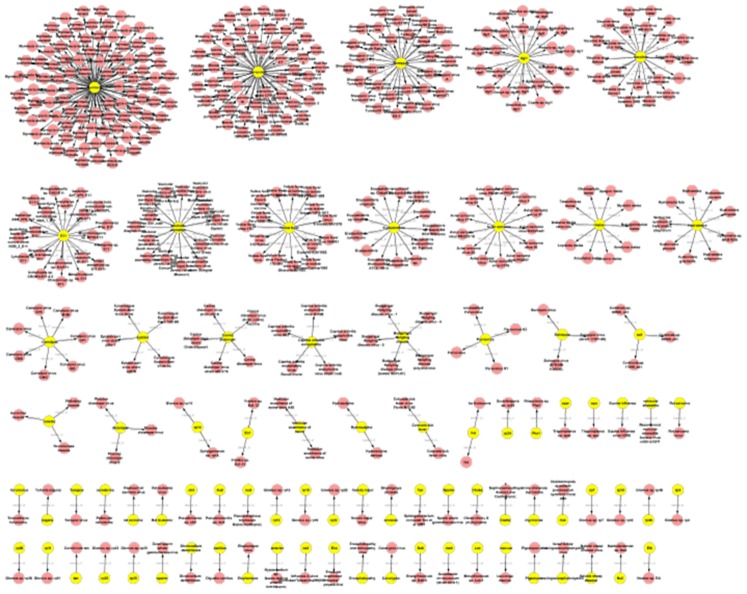
Graphs of nestedness for species terms: Terms for living beings (LIVB) contain terms of diseases but no terms of other types.

**Figure 4:**
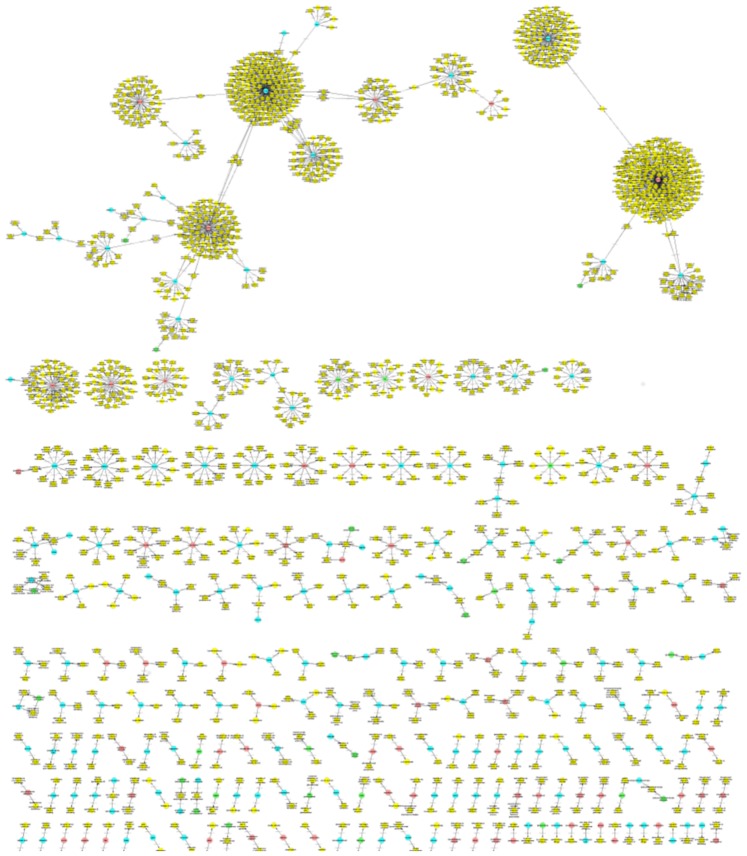
Graphs of nestedness for disease terms: Disease terms are again compositional and make use of species terms, chemical entities and protein named entities. Only a few disease terms are composed of terms of different types.

**Figure 5:**
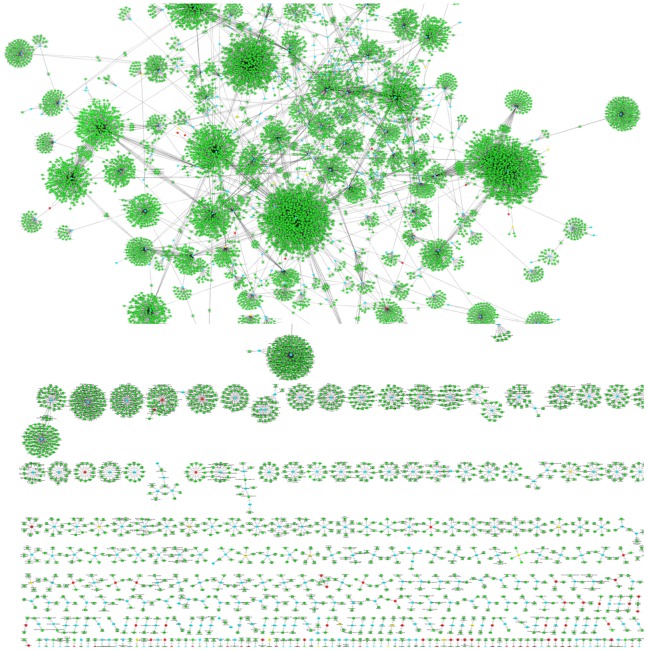
Graphs of nestedness for PGNs: The diagram gives an overview on the graphs based on those PGNs that are composed of a term of a different type. The diagram shows that a large portion of protein/gene terms contain nested terms of a chemical entity, but also species terms.

As expected the smallest number of graphs of nestedness are produced for the chemical entities (cf. fig. 2; in total 30; 21 pairs, 6 triplets), i.e. this set of graphs is very sparse. For species (cf. fig. 3) there is also a rather small number of graphs and mainly disease terms are nested in the species terms (in total 53; 24 pairs, 6 triplets, 11 with more than 10 nodes). A significantly larger number of graphs have been produced for diseases (cf. fig. 4; 520 in total; 320 pairs, 85 triplets, 15 with more than 10 nodes) and the semantic types of the nested terms are either species as well as chemical entities. The largest number graphs and the biggest graphs have been generated for PGNs (cf. fig. 5; in total 629, 291 pairs, 104 triplets, 46 with more than 10 nodes). The overview shows that different types of terms are contained and that the complexity of the PGN terminology allows for the inclusion of several nested terms leading to a complex and large graph of nestedness.

#### Considering term length of PGNs

**Figure 6:**
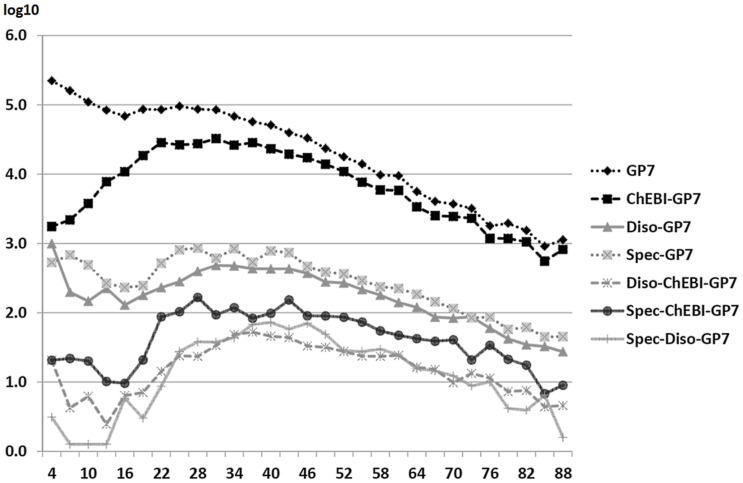
Occurrence of terms in LexEBI according to their length: The terms (baseforms and term variants) from the different resources have been matched against the GP7 terms in LexEBI. The results have been sorted according to the term length (x = 1 to 89) and the frequencies are presented in logarithmic scale (y = 0 to 6.0). After sorting, the results for the terms have been grouped into bins where each bin represents terms of a given length +/−1. For GP7 the overall occurrence is given, for the other resources the numbers indicate how many occurrences of a GP7 term contain a term of the alternative resource, e.g. ChEBI. A large portion of GP7 terms do contain ChEBI terms, and - to a lower rate - a disease or a species term. It is obvious that longer terms are more likely to be composed of terms of a different semantic type. According to the annotation guidelines, species terms should not be part of the PGN.

Fig. 6 gives an overview of the nestedness of terms according to their length in LexEBI. The diagram demonstrates the distribution of terms according to their length and the number of included terms of a different type. These figures demonstrate the amount of terms that would require special treatment in the use of Medline in any information extraction solution. A similar approach has already been tested for GO terms and has also measured the frequency of term inclusions [50].

### Distribution of Terms in Medline and BNC

In the last step of the analysis we have measured the number of terms that can be identified in Medline and the BNC. We expect that biomedical terms appear in the biomedical literature at a higher frequency and more comprehensively than in corpora for general English. Table 7 gives an overview on the distribution of the GP6 and GP7 terms across Medline and the BNC. A large portion of the enzyme terms can be identified from Medline, whereas only a small portion of the Interpro terms have been found. For the whole collection of GP6 and -7, about half of the baseforms can be extracted from the scientific literature. As expected, the same numbers are smaller when identifying the terms across the BleNC, since the BNC corpus is smaller in size. On the other side, the ratio of term variants related to Interpro and enzymes baseforms is considerably larger than on the BNC, which indicates that BNC covers different domain knowledge than Medline.

**Table 7:** Use of baseforms in Medline and BNC.

	Medline(2,180,887,571 tokens)			BNC(91,852,411 tokens)		
	Exact M.	Fuzzy M.	Ratio	Exact M.	Fuzzy M.	Ratio
GP 7.0	212′114	403′452	1.90	10′706	17′300	1.62
GP 6.0	196′390	381′665	1.94	10′073	16′455	1.63
InterPro	2′626	15′558	5.92	119	232	1.95
Enzymes	4′856	23′072	4.75	122	170	1.39
ChEBI	27′750	70′287	2.53	2′014	3′734	1.85
Species	107′797	147′106	1.36	5′374	10′181	1.89

The table gives an overview on the identification of unique terms from the different resources across the two literature repositories: Medline abstracts and the British National Corpus. The statistics counts unique terms that have been identified at least once in the two corpora.

#### Distribution of acronyms

LexEBI also provides abbreviations that have been extracted from Medline and PubmedCentral. All abbreviations have been classified to a given type and the long form of the abbreviation serves as baseform. Ta 3 gives an overview to all abbreviations.

It is expected but still remarkable, that disease acronyms, for example “AD” and “CD” for Alzheimer’s and Crohn’s Disease, respectively, and acronyms for chemical entities, for example “LPS” for Lipopolysaccharide, have the highest occurrence rates, whereas the acronyms of other semantic types have lower occurrence rates. Still the highest number of acronyms is encountered for PGNs, whereas for species only a small number of acronyms are known. For enzymes also a small number can be identified, but this small number covers almost the full domain of enzyme mentions after all.

The distribution of acronyms shows that the high diversity of entities for PGNs and species terms seems to be underrepresented and a core of chemical entity terms, enzyme terms and disease terms play an important role.

#### Distribution of nested terms across Medline

In the next step, we extracted the GP7 terms from Medline and analyzed the inclusion of terms of different semantic types in the PGNs. This approach should give new insights, how the distribution of compositional terms is across Medline, whether a minimum length to this phenomenon exists and what semantic types are more prone to form part of the PGNs. Similar information can already be derived from the cross-comparison of terms in LexEBI alone (cf. fig. 6), but we tried to identify whether the compositional terms show a different distribution than over LexEBI alone.

We distinguished the baseforms and term variants according to their length and sorted them into bins that collect terms of a given length +/−1 character difference length. We then measured the distribution of the terms across Medline and the inclusion of terms of a different type into the identified terms. In the first analysis we measured the number of occurrences of a term across Medline. As expected, the frequency of a term declines with the length of a term. The number of terms that make reference to a chemical entity is 0.5 to 1 log scale smaller than the overall number of encountered terms, i.e. at least one term out of 10 contains a term of a chemical entity. Disease and species terms can be found at a lower rate (1-2 log scales) as part of the GP7 terms along all bins containing terms of different lengths.

#### Distribution of unique terms across medline

**Figure 7:**
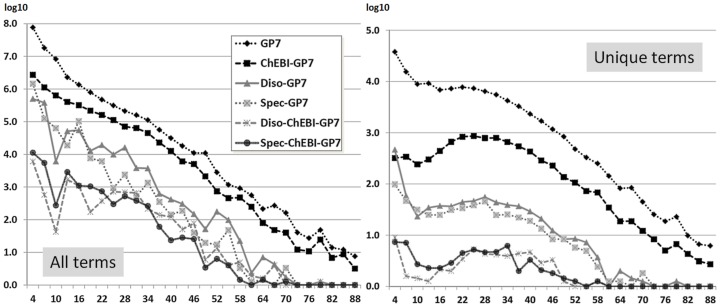
Occurrence of terms in Medline, sorted by term length: The terms (baseforms and term variants) from the different resources have been matched against Medline. The results have been sorted according to the term length and are presented in logarithmic scale (cf. fig. 6). The left diagram counts all occurrences of a GP7 term in Medline. The term lists has been manually curated to remove senseless terms with high frequencies and all occurrences of a term in a single abstract has only been counted once (“unique terms”). A large portion of GP7 terms do contain ChEBI terms, and to a lower rate a disease or a species term. For the right diagram, every GP7 term has only be counted once across all Medline. It becomes clear that longer PGNs contain mentions of chemical entities, and also species and disease terms, which both may have shared polysemous terms (very similar distribution values).

In the next step, we removed the most frequent uninformative or polysemous terms, i.e. terms with attribution to two different semantic types, from the term sets, which are mainly the terms “protein”, “ATP” and “RNA” in ChEBI and “Beta” for a species, which are frequently repeated as part of GP7 terms, but not relevant for this analysis. After removal, we again counted all occurrences, but normalized repeated occurrences in a single Medline abstract to a single count, i.e. we count Medline abstracts containing the given term (called “unique term”). This solution reduces redundancy, but still gives a representative figure for the distribution of terms across all Medline (cf. fig. 7, left diagram). We find a distribution that is similar to the previous one, but shows a more even distribution of terms across the different lengths of the terms, indicating that shorter and longer terms are used at similar frequencies, but shorter terms are used more repetitive in single Medline abstracts. Terms with a length bigger than 20 characters show higher degrees of nestedness containing chemical entities, disease or species terms, and terms with a length of less than 50 characters form the biggest portion of terms containing nested other terms.

In the next analysis, we have again normalized the results in such a way that we count an occurring term only once at all, giving an overview on the distribution of terms used in Medline that have included alternative terms (cf. fig. 7, right diagram). The diagram shows a similar distribution of terms as can be seen in the analysis across LexEBI (cf. fig. 7).

## Discussion

The Lexeome covers the terms used in the biomedical domain to describe entities. Our study gives an overview on the full set of terms from existing resources and also provides the extracted term set in a standardized format (LexEBI). The analysis illustrates how the composition of biomedical terms reflects the researchers’ ways to conceptualize their findings, in particular concerning biomedical entities. These entities play an important role, since they convey the notion of an object with some kind of existence, appearance and well-defined roles and functions and eventually serve as elements for ongoing research.

According to our findings, the baseform of PGNs from the primary data resources are less ambiguous than the term variants, which can be expected. Enzyme terms are hosted in different repositories and are popular term variants leading to the result that normalization requires additional assumptions and input for their disambiguation. PGN family terms are provided from Interpro but the differentiation to PGNs from other resources is not clear-cut inducing ambiguity between different abstraction levels (“vertical ambiguity”).

The literature analysis shows that a small portion of terms, i.e. less than 1,000, form the core of nestedness across a large number terms and that specific patterns of ambiguity can be observed, i.e. ambiguous use of species and disease terms, nestedness of chemical entities in PGNs, and vertical ambiguity. Our graph visualization based on the nestedness relation demonstrates the same result and gives an overview on the complexity of term forms for PGNs. In the case of complex text mining tasks, this complexity has to be resolved to correctly distinguish the different entity types [51]. The evaluation of terms against the scientific literature (Medline) leads to the result that only a small portion of terms are frequently used. This amount may increase when analysing the full-text literature, but large portions of the terminology may still be limited to selective and specialized use [52]. This finding partially reflects our observations that uncommon terms are included in the scientific databases, e.g. hypothetical terms, and on the other side, that term usage varies over time following the topics of the research questions under scrutiny [53], [54].

The compositional structure of GO labels has been well studied and led to the result that better term representations for the concept labels improve the quality of the ontology [8], [50]. In the case of GO, these considerations have been successful and nowadays GO includes a ‘regulate’-relation to better support compositional structures. Further research is making use of these results to improve the extraction of regulatory events from the scientific literature making use of deep semantics from the ontology [55], [56]. We can expect for the future that terminological and ontological resources will provide similar features and similar benefits to the scientific community.

In recent years, the use of ontological resources has gained importance and the amount of available ontological resources has grown to a remarkable level [57]. Often ontologies are exploited as terminological resources, but there is no doubt that ontologies serve the purpose of conceptualizing the scientific domain knowledge whereas scientific databases and terminological resources have more modest objectives [58], [59].

Ontologies are used to implement the logical framework of the domain knowledge in a formal way, whereas a terminology (or a lexicon) will only index and reference entities and - according to our analysis - define the scope of features representing entities in the biomedical scientific domain (the Lexeome), but fall short to explain the semantics.

## References

[1] Rebholz-Schuhmann D, Oellrich A, Hoehndorf R (2012) Text-mining solutions for biomedical research: enabling integrative biology. Nat Rev Genet 13: 829–839. 23150036 10.1038/nrg3337

[2] Harrow I, Filsell W, Woollard P, Dix I, Braxtenthaler M, et al.. (2012) Towards virtual knowledge broker services for semantic integration of life science literature and data sources. Drug Discovery Today. In Print. 10.1016/j.drudis.2012.11.012 23247259

[3] Rebholz-Schuhmann D, Kirsch H, Couto F (2005) Facts from text-is text mining ready to deliver? PLoS biology 3: e65. 15719064 10.1371/journal.pbio.0030065 PMC548955

[4] Thompson P, McNaught J, Montemagni S, Calzolari N, del Gratta R, et al (2011) The biolexicon: a large-scale terminological resource for biomedical text mining. BMC Bioinformatics 12: 397. 21992002 10.1186/1471-2105-12-397 PMC3228855

[5] Rebholz-Schuhmann D, Jimeno-Yepes A, Li C, Kafkas S, Lewin I, et al (2011) Assessment of ner solutions against the first and second calbc silver standard corpus. J biomedical semantics 2: S11. 10.1186/2041-1480-2-S5-S11 PMC3239301 22166494

[6] Schofield PN, Gkoutos GV, Gruenberger M, Sundberg JP, Hancock JM (2010) Phenotype ontologies for mouse and man: bridging the semantic gap. Disease models & mechanisms 3: 281–289. 20427557 10.1242/dmm.002790 PMC2860848

[7] Ogren PV, Cohen KB, Acquaah-Mensah GK, Eberlein J, Hunter L (2004) The compositional structure of Gene Ontology terms. Pac Symp Biocomput : 214–225. 10.1142/9789812704856_0021 PMC2490823 14992505

[8] Verspoor K, Dvorkin D, Cohen KB, Hunter L (2009) Ontology quality assurance through analysis of term transformations. Bioinformatics 25: i77–i84. 19478020 10.1093/bioinformatics/btp195 PMC2687949

[9] Bodenreider O, Rindesch TC, Burgun A (2002) Unsupervised, corpus-based method for extending a biomedical terminology. In: Proceedings of the ACL-02 workshop on Natural language processing in the biomedical domain-Volume 3. Association for Computational Linguistics, 53–60.

[10] Dinarelli M, Rosset S (2012) Tree representations in probabilistic models for extended named entities detection. EACL 2012 174.

[11] Roos M, Marshall MS, Gibson AP, Schuemie M, Meij E, et al (2009) Structuring and extracting knowledge for the support of hypothesis generation in molecular biology. BMC Bioinformatics 10 Suppl 10S9. 10.1186/1471-2105-10-S10-S9 PMC2755830 19796406

[12] Rzhetsky A, Iossifov I, Loh JM, White KP (2006) Microparadigms: chains of collective reasoning in publications about molecular interactions. Proc Natl Acad Sci USA 103: 4940–4945. 16543380 10.1073/pnas.0600591103 PMC1402650

[13] Clare A, Croset S, Grabmueller C, Liakata M, Oellrich A, et al.. (2011) Exploring the generation and integration of publishable scientific facts using the concept of nano-publications. In: Proceedings of the 2011 workshop on Semantic Publications at the Extended Semantic Web Conference. Hersonissos, Crete, Greece.

[14] Leitner F, Chatr-aryamontri A, Mardis SA, Ceol A, Krallinger M, et al (2010) The FEBS Letters/BioCreative II.5 experiment: making biological information accessible. Nat Biotechnol 28: 897–899. 20829821 10.1038/nbt0910-897

[15] Casher O, Rzepa HS (2006) SemanticEye: a semantic web application to rationalize and enhance chemical electronic publishing. J Chem Inf Model 46: 2396–2411. 17125182 10.1021/ci060139e

[16] Callahan A, Dumontier M, Shah NH (2011) Hyque: evaluating hypotheses using semantic web technologies. Journal of biomedical semantics 2 Suppl 2S3. 10.1186/2041-1480-2-S2-S3 PMC3102892 21624158

[17] Bodenreider O (2004) The Unified Medical Language System (UMLS): integrating biomedical terminology. Nucleic Acids Res 32: D267–270. 14681409 10.1093/nar/gkh061 PMC308795

[18] Ashburner M, Ball CA, Blake JA, Botstein D, Butler H, et al (2000) Gene ontology: tool for the unification of biology. The Gene Ontology Consortium. Nat Genet 25: 25–29. 10802651 10.1038/75556 PMC3037419

[19] Apweiler R, Martin MJ, O’Donovan C, Magrane M, Alam-Faruque Y, et al (2011) Ongoing and future developments at the Universal Protein Resource. Nucleic Acids Res 39: D214–219. 21051339 10.1093/nar/gkq1020 PMC3013648

[20] Degtyarenko K, Matos Pd, Ennis M, Hastings J, Zbinden M, et al.. (2007) ChEBI: a database and ontology for chemical entities of biological interest. Nucl Acids Res : gkm791. 10.1093/nar/gkm791 PMC2238832 17932057

[21] Maglott D, Ostell J, Pruitt KD, Tatusova T (2011) Entrez Gene: gene-centered information at NCBI. Nucleic Acids Res 39: D52–57. 21115458 10.1093/nar/gkq1237 PMC3013746

[22] Hunter S, Apweiler R, Attwood TK, Bairoch A, Bateman A, et al (2009) InterPro: the integrative protein signature database. Nucleic Acids Research 37: D211–D215. 18940856 10.1093/nar/gkn785 PMC2686546

[23] Pezik P, Jimeno-Yepes A, Lee V, Rebholz-Schuhmann D (2008) Static dictionary features for term polysemy identification. In: Building and evaluating resources for biomedical text mining, LREC Workshop.

[24] Hakenberg J, Gerner M, Haeussler M, Solt I, Plake C, et al.. (2011) The GNAT library for local and remote gene mention normalization. Bioinformatics. 10.1093/bioinformatics/btr455 PMC3179658 21813477

[25] Wermter J, Tomanek K, Hahn U (2009) High-performance gene name normalization with GeNo. Bioinformatics. 25: 815–821. 10.1093/bioinformatics/btp071 19188193

[26] Tsuruoka Y, McNaught J, Ananiadou S (2008) Normalizing biomedical terms by minimizing ambiguity and variability. BMC Bioinformatics 9: S2. 10.1186/1471-2105-9-S3-S2 PMC2352870 18426547

[27] Spasic I, Schober D, Sansone SA, Rebholz-Schuhmann D, Kell DB, et al (2008) Facilitating the development of controlled vocabularies for metabolomics technologies with text mining. BMC bioinformatics 9: S5. 10.1186/1471-2105-9-S5-S5 PMC2367623 18460187

[28] Waagmeester A, Pezik P, Coort S, Tourniaire F, Evelo C, et al (2009) Pathway enrichment based on text mining and its validation on carotenoid and vitamin a metabolism. Omics : a journal of integrative biology 13: 367–379. 19715393 10.1089/omi.2009.0029

[29] Rebholz-Schuhmann D, Arregui M, Gaudan S, Kirsch H, Jimeno A (2008) Text processing through web services: calling whatizit. Bioinformatics (Oxford, England) 24: 296–298. 10.1093/bioinformatics/btm557 18006544

[30] Kirsch H, Gaudan S, Rebholz-Schuhmann D (2006) Distributed modules for text annotation and ie applied to the biomedical domain. International journal of medical informatics 75: 496–500. 16085453 10.1016/j.ijmedinf.2005.06.011

[31] Rinaldi F, Kaljurand K, Saetre R (2011) Terminological resources for text mining over biomedical scientific literature. Artif Intell Med 52: 107–114. 21652190 10.1016/j.artmed.2011.04.011

[32] Liu H, Hu ZZ, Zhang J, Wu C (2006) Biothesaurus: a web-based thesaurus of protein and gene names. Bioinformatics (Oxford, England) 22: 103–105. 10.1093/bioinformatics/bti749 16267085

[33] Smith B, Ashburner M, Rosse C, Bard J, Bug W, et al (2007) The OBO Foundry: coordinated evolution of ontologies to support biomedical data integration. Nat Biotechnol 25: 1251–1255. 17989687 10.1038/nbt1346 PMC2814061

[34] Hettne KM, Stierum RH, Schuemie MJ, Hendriksen PJM, Schijvenaars BJA, et al (2009) A dictionary to identify small molecules and drugs in free text. Bioinformatics 25: 2983–2991. 19759196 10.1093/bioinformatics/btp535

[35] Miller GA (1995) Wordnet: a lexical database for english. Communications of the ACM 38: 39–41.

[36] Navigli R, Ponzetto SP (2012) Babelnet: The automatic construction, evaluation and application of a wide-coverage multilingual semantic network. Artificial Intelligence.

[37] Rebholz-Schuhmann D, Kafkas S, Kim JH, Yepes AJ, Hoehndorf R, et al.. (2013) Performance analysis of different protein/gene tagging solutions against public gold standard corpora. (Submitted).

[38] de Matos P, Alcantara R, Dekker A, Ennis M, Hastings J, et al (2010) Chemical Entities of Biological Interest: an update. Nucleic Acids Res 38: D249–254. 19854951 10.1093/nar/gkp886 PMC2808869

[39] Fleischmann A, Darsow M, Degtyarenko K, Fleischmann W, Boyce S, et al (2004) IntEnz, the integrated relational enzyme database. Nucleic Acids Res 32: D434–437. 14681451 10.1093/nar/gkh119 PMC308853

[40] Du P, Feng G, Flatow J, Song J, Holko M, et al (2009) From disease ontology to disease-ontology lite: statistical methods to adapt a general-purpose ontology for the test of gene-ontology associations. Bioinformatics 25: i63–68. 19478018 10.1093/bioinformatics/btp193 PMC2687947

[41] Bodenreider O, Zhang S (2006) Comparing the representation of anatomy in the FMA and SNOMED CT. AMIA Annu Symp Proc : 46–50. PMC1839313 17238300

[42] Apache Lucene Core Project Web site. Available: http://lucene.apache.org/java/docs/index.html. Accessed 2013 Sep 3.

[43] Aronson AR, Lang FM (2010) An overview of MetaMap: historical perspective and recent advances. J Am Med Inform Assoc 17: 229–236. 20442139 10.1136/jamia.2009.002733 PMC2995713

[44] McEntyre JR, Ananiadou S, Andrews S, Black WJ, Boulderstone R, et al (2011) Ukpmc: a full text article resource for the life sciences. Nucleic Acids Res 39: D58–D65. 21062818 10.1093/nar/gkq1063 PMC3013671

[45] Schwartz AS, Hearst MA (2003) A simple algorithm for identifying abbreviation definitions in biomedical text. Pacific Symposium on Biocomputing : 451–462. 12603049

[46] Kuo CJ, Ling M, Lin KT, Hsu CN (2009) Bioadi: a machine learning approach to identifying abbreviations and definitions in biological literature. BMC bioinformatics 10: S7. 10.1186/1471-2105-10-S15-S7 PMC2788358 19958517

[47] Sohn S, Comeau DC, Kim W, Wilbur WJ (2008) Abbreviation definition identification based on automatic precision estimates. BMC bioinformatics 9: 402. 18817555 10.1186/1471-2105-9-402 PMC2576267

[48] Yeganova L, Comeau DC, Wilbur WJ (2011) Machine learning with naturally labeled data for identifying abbreviation definitions. BMC bioinformatics 12: S6. 10.1186/1471-2105-12-S3-S6 PMC3111592 21658293

[49] Kim JJ, Pezik P, Rebholz-Schuhmann D (2008) Medevi: retrieving textual evidence of relations between biomedical concepts from medline. Bioinformatics (Oxford, England) 24: 1410–1412. 10.1093/bioinformatics/btn117 PMC2387223 18400773

[50] Ogren PV, Cohen KB, Hunter L (2005) Implications of compositionality in the gene ontology for its curation and usage. In: Pacific Symposium on Biocomputing. p. 174. 15759624

[51] Rinaldi F, Clematide S, Garten Y, Whirl-Carrillo M, Gong L, et al (2012) Using ODIN for a PharmGKB revalidation experiment. Database (Oxford) 2012: bas021. 22529178 10.1093/database/bas021 PMC3332569

[52] Bada M, Eckert M, Evans D, Garcia K, Shipley K, et al (2012) Concept Annotation in the CRAFT corpus. BMC Bioinformatics 13: 161. 22776079 10.1186/1471-2105-13-161 PMC3476437

[53] Hoffmann R, Valencia A (2003) Life cycles of successful genes. Trends Genet 19: 79–81. 12547515 10.1016/s0168-9525(02)00014-8

[54] Gaudan S, Kirsch H, Rebholz-Schuhmann D (2005) Resolving abbreviations to their senses in medline. Bioinformatics (Oxford, England) 21: 3658–3664. 10.1093/bioinformatics/bti586 16037121

[55] Beisswanger E, Lee V, Kim JJ, Rebholz-Schuhmann D, Splendiani A, et al (2008) Gene regulation ontology (gro): design principles and use cases. Studies in health technology and informatics 136: 9–14. 18487700

[56] Kim J, Rebholz-Schuhmann D (2011) Improving the extraction of complex regulatory events from scientific text by using ontology-based inference. J biomedical semantics 2: S3. 10.1186/2041-1480-2-S5-S3 PMC3239303 22166672

[57] Hoehndorf R, Dumontier M, Gkoutos GV (2012) Evaluation of research in biomedical ontologies. Brief Bioinformatics. 10.1093/bib/bbs053 PMC3888109 22962340

[58] Gruber TR (1993) A translation approach to portable ontology specifications. Knowledge acquisition 5: 199–220.

[59] Gaudan S, Yepes AJ, Lee V, Rebholz-Schuhmann D (2008) Combining evidence, specificity, and proximity towards the normalization of gene ontology terms in text. EURASIP journal on bioinformatics and systems biology : 3427–46. 10.1155/2008/342746 PMC3171395 18437221

